# Adenomatoid odontogenic tumor in an unusual posterior maxillary location: a rare case report in a young male

**DOI:** 10.3389/froh.2025.1734444

**Published:** 2025-12-17

**Authors:** Natalí González, Estefanía Chávez-Mestanza, Náthaly Mercedes Román-Galeano, Claudia Reytor-González, Daniel Simancas-Racines

**Affiliations:** 1 Universidad UTE, Facultad de Odontología, Santo Domingo, Ecuador; 2 Facultad de Ciencias Médicas, de la Salud y la Vida, Universidad Internacional del Ecuador UIDE, Quito, Ecuador; 3 Escuela de Medicina, Pontificia Universidad Católica del Ecuador, Santo Domingo, Ecuador; 4 Facultad de Ciencias de la Salud y Bienestar, Pontificia Universidad Católica del Ecuador, Quito, Ecuador

**Keywords:** adenomatoid odontogenic tumor, ameloblastoma, odontogenic tumor, oral surgical procedures, case reports

## Abstract

The adenomatoid odontogenic tumor is a rare benign epithelial odontogenic neoplasm that most frequently affects young women and typically occurs in the anterior maxilla. Its presentation in the posterior maxilla, especially in male patients, is uncommon and can create diagnostic challenges. This case describes a large posterior maxillary adenomatoid odontogenic tumor in a 16-year-old male who presented with a one-year history of progressive, painless swelling of the right cheek. Clinical examination revealed facial asymmetry, obliteration of the right nasolabial fold, and intraoral swelling extending from tooth 1.5 to the posterior maxilla. Panoramic radiography and computed tomography showed a multilocular radiolucent lesion with a “soap bubble” appearance, internal calcifications, and displacement of tooth 1.8 toward the floor of the right orbit, which remained intact. The lesion caused root resorption of adjacent teeth and extensive destruction of the maxillary bone. Surgical treatment consisted of enucleation and extraction of teeth 1.5–1.8, followed by histopathological confirmation of adenomatoid odontogenic tumor. Due to the degree of bone involvement, a subsequent wide resection of critical maxillofacial structures was necessary. Postoperative follow-up at five months showed no recurrence but significant residual anatomical changes. This case emphasizes the importance of including adenomatoid odontogenic tumor in the differential diagnosis of posterior maxillary lesions in male patients, and the need for careful surgical planning, histopathological confirmation, and long-term follow-up.

## Introduction

Adenomatoid odontogenic tumor (AOT) is a benign epithelial odontogenic neoplasm characterized by slow growth, well-defined borders, and an excellent prognosis. Although uncommon, it is a well-recognized entity within the spectrum of odontogenic tumors and typically presents as an incidental radiographic finding or a painless swelling discovered during routine dental evaluation. Because AOT may share clinical and radiographic features with other odontogenic lesions, accurate diagnosis relies on correlating imaging with histopathological examination (1).

AOT most frequently affects female patients in their second decade of life and shows a strong predilection for the anterior maxilla, where it is often associated with an unerupted canine. Lesions are usually small—commonly less than 3 cm—, asymptomatic, and exhibit unilocular radiolucency with occasional fine calcifications. These characteristic features are well documented and represent the classic presentation of the tumor. Notably, posterior maxillary involvement, male patients, large lesions, and destructive patterns are distinctly uncommon, making such cases clinically significant due to their deviation from the classical epidemiological and anatomical profile (2).

In this report, we describe an unusually extensive AOT occurring in the posterior maxilla of a young male patient, exhibiting aggressive-appearing radiographic features, extensive bone involvement, and significant tooth displacement. This atypical presentation required a broader diagnostic approach and a more complex surgical plan than typically indicated for AOTs. The present case contributes to the limited literature on posterior maxillary AOTs and underscores the importance of recognizing variations in location, size, and biological behavior to guide appropriate clinical management.

## Case presentation

A 16-year-old mestizo male, with no relevant medical history, presented with a one-year history of progressive, painless swelling of the right cheek. The patient reported gradual enlargement of the swelling over the previous year.

## Clinical findings

Physical examination revealed marked facial asymmetry, obliteration of the right nasolabial fold, and a prominent bulge in the right nasomaxillary region. Intraorally, there was a firm swelling of the right maxilla, extending from the left lateral incisor (tooth 2.2) to the posterior third of the maxilla in quadrant 1, with asymmetry in the right half of the palate. There were no signs of inflammation, and palpation was painless.

## Diagnostic assessment

**Figure 1:**
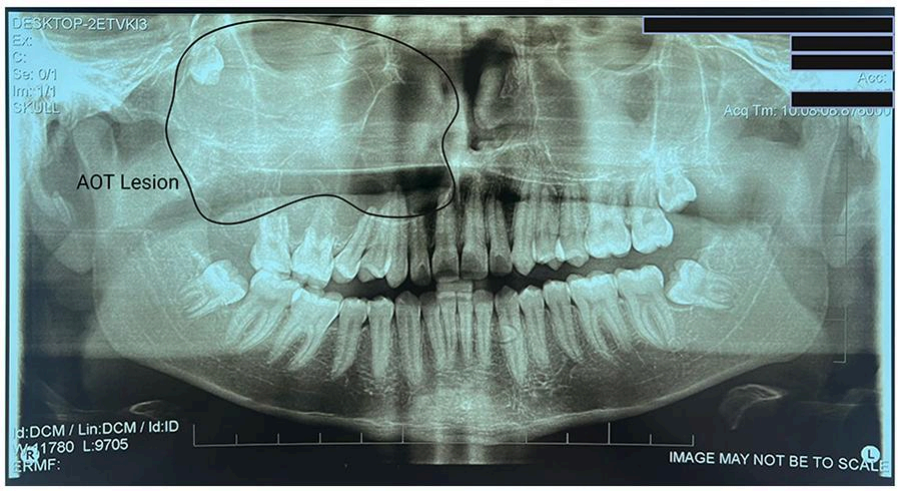
Preoperative panoramic radiograph of the maxilla and mandible. Radiograph showing a multilocular radiolucent lesion with internal calcifications in the right hemimaxilla, involving teeth 1.5, 1.6, 1.7, and 1.8. The lesion causes displacement of the third molar (1.8) toward the floor of the right orbit, which remains intact. It exhibits a “soap bubble” appearance and well-defined borders.

**Figure 2:**
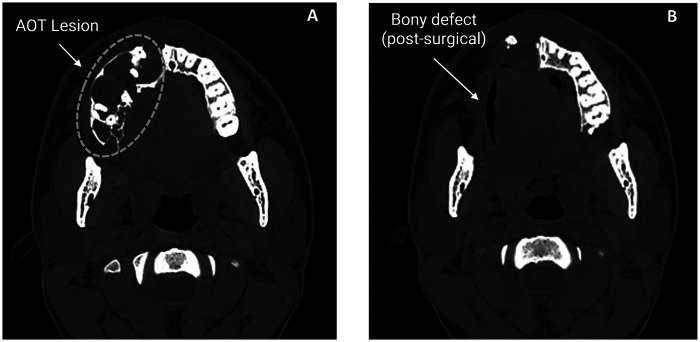
Axial computed tomography images. **(A)** Preoperative scan showing a multilocular lesion in the right hemimaxilla with a “soap bubble” appearance and internal calcifications (outlined in green), involving teeth 1.5, 1.6, 1.7, and 1.8, and extending across the midline to involve the left maxilla. **(B)** Postoperative scan at 5-month follow-up demonstrating absence of the lesion and postoperative changes in the right maxillary region.

Panoramic radiography demonstrated a well-defined radiolucent lesion in the right hemimaxilla with a “soap bubble” appearance and internal calcifications, associated with displacement of the third molar (tooth 1.8) (Figure 1). Computed tomography revealed a multilocular “soap bubble” lesion occupying the right hemimaxilla and crossing the midline to involve the left maxilla. The right maxillary sinus was filled up to the orbital floor, which remained intact (Figure 2A). There were destruction of the right palatal bone and the right maxilla, sparing only the nasomaxillary aspect. Root resorption was present in teeth 1.6 and 1.7. The lesion expanded the bone with thinning and partial loss of cortical plates in certain areas. The third molar (1.8) was displaced toward the roof of the right maxillary sinus.

Although dentigerous cyst and odontogenic keratocyst were initially considered among the diagnostic possibilities, a more detailed evaluation of the imaging studies revealed features that were inconsistent with cystic lesions. The mixed radiolucent–radiopaque pattern, presence of internal calcifications, and associated root resorption strongly suggested a solid odontogenic tumor rather than a fluid-filled cavity. For this reason, the final differential diagnosis focused on odontogenic tumors, with desmoplastic ameloblastoma and calcifying epithelial odontogenic tumor representing the most compatible entities based on the radiographic characteristics observed. The ICD-10 code assigned was D10.3—Benign neoplasm of other and unspecified parts of the mouth.

## Therapeutic intervention

The surgical approach consisted of enucleation of the lesion with extraction of teeth 1.5, 1.6, 1.7, and 1.8. Biopsy specimens were obtained from the tumor mass and from the surgical margins after enucleation to confirm tumor-free borders. Gross examination revealed a solid tumor measuring approximately 40 mm in diameter, covered by a fibrous capsule and containing the unerupted tooth 1.8 within the tissue.

No aspiration puncture was performed because the radiographic features—internal calcifications, mixed density, and root resorption—were not compatible with a cystic lesion and made aspiration unlikely to provide diagnostic value. Therefore, the surgical team proceeded with complete enucleation and extraction in a single procedure.

Histological examination demonstrated a myxomatous stroma containing multiple cyst-like structures of varying sizes, lined by two or more layers of simple cuboidal cells, with lumens filled with amorphous eosinophilic material. These structures were accompanied by congested blood vessels and cells with clear cytoplasm and peripheral nuclei, along with bone spicules. The tumor capsule consisted mainly of hyalinized fibroconnective tissue lined by cuboidal epithelial cells, containing gland-like acini and a mononuclear inflammatory infiltrate. Odontogenic epithelium surrounding a stellate reticulum was also observed. The histopathological diagnosis confirmed an AOT.

## Follow-up and outcomes

The immediate postoperative course was favorable, with no signs of infection or early recurrence. At five months, however, follow-up computed tomography revealed hypodense lesions in the maxillary sinus and nasomaxillary wall (Figure 2B). A second surgical procedure was performed to rule out recurrence or residual tumor. Histopathological analysis revealed nasal polyps lined by respiratory and stratified epithelium, with an edematous corium containing mixed inflammatory infiltrate, congested vessels, and bone spicules.

Due to the extensive bone destruction caused by the primary lesion, a wide resection of maxillofacial structures was required, including the nasal septum, vomer, inferior portion of the ethmoid bone, and the left nasomaxillary wall, in addition to the right posterior maxilla and part of the hard palate. This resulted in a significant residual anatomical defect, which, although not immediately compromising vital functions, necessitates ongoing monitoring due to its aesthetic, functional, and psychological implications.

## Discussion

AOT is a benign epithelial odontogenic neoplasm with excellent prognosis. However, its diagnosis can be challenging when it arises in atypical anatomical locations or in patients who do not fit the classical epidemiological profile, which is predominantly young females in their second decade of life (3). Moreover, its slow growth and well-circumscribed radiographic appearance may mimic other odontogenic lesions, including ameloblastoma or calcifying odontogenic tumors, potentially leading to overtreatment if histopathological confirmation is not obtained (4).

In the present case, the tumor occurred in a young male patient, with an unusual posterior maxillary location and an expansive growth pattern that clinically simulated a more aggressive neoplasm. The lesion reached a considerable size, causing tooth displacement, root resorption, and extensive maxillary bone destruction. These features necessitated a more extensive surgical approach than is typical for AOTs, which are usually amenable to conservative enucleation (5).

Posterior maxillary AOTs are rare, but several published cases allow meaningful comparison with our findings. John et al. reported an AOT associated with a dentigerous cyst involving an impacted second molar in the posterior maxilla of a 39-year-old woman, producing sinus displacement but preserving cortical integrity (6). Rao et al. described a posterior maxillary AOT in a 17-year-old male, also associated with a molar, which expanded toward the maxillary sinus without destructive behavior (7). Assao et al. documented a posterior maxillary AOT associated with an odontoma, presenting as a mixed radiolucent–radiopaque lesion with well-defined borders and no aggressive extension into adjacent structures (8). Compared with these reports, our case demonstrated a more destructive pattern of involvement, including root resorption, thinning of the nasomaxillary and palatal walls, and extension toward the nasal cavity and ethmoid region. These differences suggest that although posterior maxillary AOTs generally maintain the benign, encapsulated behavior characteristic of this tumor, certain cases—such as ours—may present with increased expansiveness potentially related to anatomical constraints, delayed diagnosis, or early onset during active craniofacial growth.

Although the initial enucleation was successful, the significant structural compromise caused by the lesion required subsequent resection of critical anatomical structures, including the nasal septum, vomer, portions of the ethmoid bone, and the left nasomaxillary wall, in addition to the right posterior maxilla and part of the hard palate. Such a degree of tissue loss is uncommon in AOTs, highlighting the importance of individualized surgical planning and the involvement of a multidisciplinary team comprising oral and maxillofacial surgeons, otolaryngologists, radiologists, and reconstructive specialists (9). This approach not only facilitates complete tumor removal but also anticipates potential reconstructive needs and addresses the psychosocial impact of residual defects.

Following the wide resection, a staged reconstructive and prosthetic rehabilitation plan is anticipated. At this phase, no reconstructive procedures have been initiated, but long-term management will require coordinated evaluation with maxillofacial prosthetics and reconstructive surgery teams. Periodic assessment of healing, functional adaptation, and craniofacial growth will determine the ideal timing for definitive rehabilitation. This approach ensures that future reconstruction or prosthetic replacement can be appropriately adapted to the patient's anatomical development, functional needs, and esthetic considerations.

Furthermore, given the patient's age and the ongoing craniofacial growth, long-term follow-up with periodic imaging is critical to detect possible recurrence, monitor healing dynamics, and assess the functional adaptation of the affected structures (10). The use of cone-beam computed tomography in follow-up may provide detailed three-dimensional evaluation while minimizing radiation exposure, an important consideration in adolescent patients. Early integration of prosthetic or surgical reconstruction planning into follow-up care can also improve functional and aesthetic outcomes.

The present report is limited by a relatively short follow-up period of five months. Longer clinical and radiographic monitoring is essential to confirm the stability of healing, assess functional adaptation, and reliably exclude delayed recurrence, particularly in a growing patient and following an extensive maxillary resection.

## Patient perspective

Proper surgical planning and rigorous clinical follow-up led to a favorable prognosis with no evidence of tumor recurrence. “When they told me I had a tumor, I was scared. The word tumor sounds very strong, and although the dentist explained that it wasn't malignant, my mind had already gone to ugly places. The most important thing for me was that they explained what I had clearly, that I felt supported, and that I wasn't afraid to ask for help when I needed it. Now I'm back in school, playing sports again, and grateful that everything went well.”

## Conclusion

This case documents a rare presentation of an AOT located in the right posterior maxilla of a young male patient—an epidemiologically and anatomically atypical scenario. The lesion's large size, aggressive-appearing radiographic features, and extensive bone destruction necessitated a broader surgical approach than is commonly required for AOTs.

Through comprehensive diagnostic assessment—including clinical, radiographic, and histopathological evaluation—and a tailored surgical plan, complete tumor removal was achieved with no evidence of recurrence during early follow-up. The case underscores the need for meticulous surgical planning, a multidisciplinary treatment approach, and long-term postoperative monitoring, especially in younger patients with significant anatomical alterations.

In addition to clinical management, attention to the patient's functional rehabilitation and psychosocial well-being is paramount. Early involvement of reconstructive and psychological support services can optimize long-term outcomes and quality of life. This report adds to the limited literature on posterior maxillary AOTs and reinforces the importance of histopathological confirmation to guide appropriate treatment, avoiding both undertreatment and unnecessary aggressive interventions.

## Data Availability

The original contributions presented in the study are included in the article/Supplementary Material, further inquiries can be directed to the corresponding authors.
